# A Multilevel Study of Eupatorin and Scutellarein as Anti-Amyloid Agents in Alzheimer’s Disease

**DOI:** 10.3390/biomedicines11051357

**Published:** 2023-05-04

**Authors:** Aikaterini E. I. Rizou, Georgia I. Nasi, Yiorgos Paikopoulos, Dimitra S. Bezantakou, Konstantina D. Vraila, Panagiotis M. Spatharas, Virginia D. Dimaki, Nikos C. Papandreou, Fotini N. Lamari, Niki Chondrogianni, Vassiliki A. Iconomidou

**Affiliations:** 1 Section of Cell Biology and Biophysics, Department of Biology, School of Sciences, National and Kapodistrian University of Athens, Panepistimiopolis, 15701 Athens, Greece; rizoukat@biol.uoa.gr (A.E.I.R.); gnasi@biol.uoa.gr (G.I.N.); dimbez@biol.uoa.gr (D.S.B.); vrailakon@biol.uoa.gr (K.D.V.); panspatharas@biol.uoa.gr (P.M.S.); npapand@biol.uoa.gr (N.C.P.); 2 Institute of Chemical Biology, National Hellenic Research Foundation, 48 Vassileos Constantinou Ave., 11635 Athens, Greece; george_paik@hotmail.com (Y.P.); nikichon@eie.gr (N.C.); 3 Laboratory of Pharmacognosy & Chemistry of Natural Products, Department of Pharmacy, University of Patras, 26504 Rion, Greece; virnadimaki@upatras.gr (V.D.D.); flam@upatras.gr (F.N.L.)

**Keywords:** Alzheimer’s disease, amyloid beta peptide, natural products, eupatorin, scutellarein, model organism, *Caenorhabditis elegans*, molecular dynamics

## Abstract

Today, Alzheimer’s disease (AD)—the most common neurodegenerative disorder, which affects 50 million people—remains incurable. Several studies suggest that one of the main pathological hallmarks of AD is the accumulation of abnormal amyloid beta (Aβ) aggregates; therefore, many therapeutic approaches focus on anti-Aβ aggregation inhibitors. Taking into consideration that plant-derived secondary metabolites seem to have neuroprotective effects, we attempted to assess the effects of two flavones—eupatorin and scutellarein—on the amyloidogenesis of Aβ peptides. Biophysical experimental methods were employed to inspect the aggregation process of Aβ after its incubation with each natural product, while we monitored their interactions with the oligomerized Aβ through molecular dynamics simulations. More importantly, we validated our in vitro and in silico results in a multicellular organismal model—namely, *Caenorhabditis elegans*—and we concluded that eupatorin is indeed able to delay the amyloidogenesis of Aβ peptides in a concentration-dependent manner. Finally, we propose that further investigation could lead to the exploitation of eupatorin or its analogues as potential drug candidates.

## 1. Introduction

Over the past decade, Alzheimer’s disease (AD), along with dementia, has been classified as the seventh leading cause of death, according to the World Health Organization [1]. AD is characterized as an age-related neurodegenerative disorder with detrimental consequences for memory and cognitive abilities that eventually leads to death. It is widely accepted that one of the pathological hallmarks of the disease at a cellular level is the presence of senile plaques in the brain, which mainly consist of amyloid fibrils [2]. The main component of these extracellular deposits is the amyloid beta (Aβ) peptide, which is derived from the proteolytic degradation of amyloid precursor protein (APP). APP is a transmembrane protein that typically participates in synaptogenesis, neurite growth, and neuronal adhesion [3]. The predominant amyloid cascade hypothesis states that proteolytic enzymes called β- and γ-secretases process APP and release the neurotoxic Aβ peptide. Aβ peptides function as monomers, aggregate into oligomers and, finally, self-assemble into mature amyloid fibrils [4]. In recent years, this theory has been vigorously challenged by several in vitro and in vivo studies suggesting that Aβ oligomers are a more toxic structure compared to amyloid fibrils. Oligomers appear to play a role in triggering neuronal dysfunction, oxidative stress, and chronic neuroinflammation [5]. Thus, a vicious circle is eventually established in which chronic inflammation and Aβ pathology fuel one another in a continuous cycle. Even though brain inflammation appears to play a neuroprotective role, such a persistent immune response could provoke neurodegeneration and accelerate amyloidogenesis [6]. Therefore, not only amyloid deposition, but also oxidative stress and inflammation are considered to be important factors in the pathogenesis of AD [7]. 

Unfortunately, despite decades of research and clinical trials, neither disease-modifying drugs nor biomarkers for early diagnosis of this lethal disease exist. According to the Alzheimer’s Association, the FDA-approved drugs that aim to decelerate the disease’s progression are categorized into three main categories: drugs that may delay the clinical decline, drugs that treat the cognitive symptoms, and drugs that treat non-cognitive symptoms (Alzheimer’s Association, 2021). The first FDA-approved drug that seems to lessen the clinical symptoms is aducanumab (Aduhelm™), which is described as an anti-amyloid antibody therapy targeting amyloid plaques. In addition, glutamate regulators and cholinesterase inhibitors are prescribed to ameliorate memory and thinking, in combination with drugs that counteract behavioral or psychological modifications. However, there is still a long way to go before treatments focus on the etiological mechanisms of AD. In recent years, the scientific community has investigated novel strategies to combat Aβ aggregation. For instance, peptides, antibodies, metal chelators, and natural products derived from plants have been evaluated in vitro and in vivo [8]. In the last decade, the multifactorial nature of the disease has led to the search for “multi-target-directed ligands”, i.e., natural or synthetic compounds with more than one complementary biological activity and a good pharmacokinetic profile [9,10]. According to this innovative approach, multifunctional agents possessing anti-inflammatory and anti-oxidative activities—in addition to anti-amyloid capacity—need to be further explored. Intensive research on tacrine and donepezil derivatives has been conducted to this end [11].

Flavones are a category of polyphenols found in many fruits and medicinal plants that, depending on the substitution, may exhibit antioxidant, anti-inflammatory, metal-chelating, and neuroprotective properties, or may also inhibit β-secretase, cholinesterases, and monoamine oxidases, as well as inhibiting Aβ aggregation and/or tau hyperphosphorylation [12,13]. Thus, they are good starting points for the development of “multi-target directed ligands” through studies of structure–activity relationships. In order to effectively inhibit monoamine oxidases and cholinesterases, substitutions of hydroxyl and/or methoxy at positions 5 and 7 of their structure are thought to be advantageous; meanwhile, a catechol group is thought to be advantageous to boost the reactive-oxygen-species-scavenging, metal-ion-chelating, and possibly Aβ-aggregation-inhibitory activity [12].

Concerning Aβ aggregation, polyphenols such as 3-epigallocatechin gallate [14], resveratrol [15], and a wide variety of flavones have shown promising results in inhibiting amyloid or amyloid-like fibril formation. The ability of flavones to inhibit fibril formation is largely determined by their substitutions, with a greater number of hydroxyl substitutions generally corresponding to a higher inhibitory potential [16]. Additionally, the absence of a hydroxyl group at position 3 seems necessary for fibril inhibition [16]. Scutellarein and eupatorin are two flavones that have a limited distribution among plant species and share substitutions at positions 5, 6, and 7 of the A-ring. Scutellarein has three hydroxy groups at those positions, whereas eupatorin has a hydroxyl group at position 5 and two methoxy groups at the other positions. Furthermore, scutellarein has one hydroxyl group at position 4′, whereas eupatorin has one hydroxyl at 3′ and one methoxy at 4′ of the B-ring (Figure S1, Supplementary File). 

Previously, scutellarein has been investigated for its neuroprotective, anti-inflammatory, and antioxidant properties [17,18]. Furthermore, in an earlier study by Malisauskas et al. [16], scutellarein (5,6,7,4′-tetrahydroxyflavone) was the best flavone inhibitor of insulin fibril formation. In contrast, eupatorin is known for its anti-inflammatory action, but it has never been tested against Aβ_42_ amyloidogenesis or AD [19]. These properties could possibly make them the ideal candidates for a multiple-target strategy.

In the current study, we conducted a comprehensive investigation using a multilevel approach encompassing in vitro, in silico, and in vivo methods to evaluate the ability of the two flavones—scutellarein and eupatorin—to inhibit or delay the Aβ_42_ aggregation. While scutellarein has shown promising results in previous studies, we aimed at uncovering the interactions between the compound and the toxic oligomeric structure of Aβ_42_. Considering the structural similarity of eupatorin to scutellarein and the fact that it has not been studied before, we investigated the anti-amyloid profile of the former in vitro, in vivo, and in silico. First, we predicted their drug-likeness in silico, using SwissADME [20], and then we utilized biophysical experimental methods to study the effects of these natural products on Aβ_42_ fibril formation. The compounds were subjected to molecular docking studies in conjunction with molecular dynamics (MD) simulations with an oligomeric structure of Aβ_42_ to understand their interactions. Specific strains of *Caenorhabditis elegans* that overexpress the human Aβ were used to confirm the in vitro results. *C. elegans* has several advantages as a model organism, including its high degree of conservation with humans; thus, it is a valuable tool for studying neurodegenerative disorders at a complex, multicellular level [21]. This study demonstrates that eupatorin and scutellarein could be further examined as potential drug candidates against AD.

## 2. Materials and Methods

### 2.1. Peptide Synthesis and Disaggregation

Aβ_42_ peptide (DAEFRHDSGYEVHHQKLVFFAEDVGSNKGAIIGLMVGGVVIA) was produced and lyophilized by GeneCust© (Boynes, France), and the purity exceeded 95%, with the N- and C-termini being free. The peptide was dissolved in 1,1,1,3,3,3-hexafluoro-2-propanol (HFIP) (Sigma©, St. Louis, MO, USA) at a concentration of 1 mg/mL to disaggregate any secondary structures formed during synthesis. The peptide solution was evaporated overnight at room temperature until a peptide-containing film was formed at the bottom of the Eppendorf tube. The peptide-containing films were stored at −20 °C. Just prior to use, each peptide-containing film was left at room temperature for 30 min.

### 2.2. The Natural Products

Scutellarein (C_15_H_10_O_6_, CAS number 529-53-3, product code FS32912) and eupatorin (C_18_H_16_O_7_, CAS number 855-96-9, product code FE65553) were bought from Carbosynth Limited (Compton, Berkshire, UK). Both were dissolved in dimethyl sulfoxide (DMSO) at the maximal solubility of 10 mg/mL.

### 2.3. Sample Preparation

Considering relevant studies, as well as the maximum solubility of each compound in DMSO, three different concentrations of the natural products were tested [22]. The Aβ_42_-peptide-containing films were mixed separately with the solutions of the natural products at three different molar ratios—1 Aβ_42_:1 natural product, 1 Aβ_42_:5 natural product and 1 Aβ_42_:10 natural product. Individual Aβ_42_ solution was used as a control for all in vitro experimental assays. Depending on the experimental assay performed, the final peptide concentration was 10 μM or 40 μM. The final concentration of DMSO from the solutions of the natural products did not exceed 3%, so as to avoid altering the Aβ_42_ aggregation kinetics [23]. The samples were placed in a 35 °C ultrasound water bath for 60 s; 4-(2-hydroxyethyl)-1-piperazineethanesulfonic acid (HEPES) (Sigma©, St. Louis, MO, USA) at a 0.1 M concentration and pH 7.5 was added to the final volume, and the samples were agitated at 37 °C for two hours. Finally, we incubated the natural product mixtures and the control at 37 °C for at least seven days.

### 2.4. Negative Staining and Transmission Electron Microscopy

The amyloid fibril formation of the Aβ_42_ peptide in the presence and absence of eupatorin and scutellarein was studied using transmission electron microscopy (TEM). A 10 μL droplet of each 40 μM sample was placed on 400-mesh glow-discharged and carbon-coated copper TEM grids for 20 min. The grids were placed on a droplet of 2% (*w*/*v*) aqueous uranyl acetate for 50 s and then washed successively with three distilled water droplets to remove the extra stain. The excess water was removed with Whatman filter paper, and the grids were left to air-dry for a few seconds. The samples were inspected using a Philips Morgagni^TM^ 268 Transmission Electron Microscope (FEI, Hillsboro, OR, USA) operated at 80 kV. Digital acquisitions were performed with an 11 megapixel side-mounted Morada CCD camera (Soft Imaging System, Muenster, Germany). The analysis of the electron micrographs was performed by ImageJ (National Institutes of Health (NIH), Bethesda, MD, USA) [24].

### 2.5. Congo Red Staining and Polarized Microscopy

A droplet from each 40 μM sample was placed on a glass slide and air-dried at room temperature. The sample was stained using 0.01 M Congo red (Sigma©, St. Louis, MO, USA) solution in PBS (137 mmol/L NaCl, 27 mmol/L KCl, 100 mmol/L Na_2_HPO_4_, 18 mmol/L KH_2_PO_4_, pH = 7.4) for approximately 20 min and washed several times with 90% ethanol. Then, the samples were left to air-dry for approximately 10 min and observed under bright-field illumination and between crossed polars, using a Leica MZ7.5 polarizing stereomicroscope (Leica Camera AG, Weltzar, Germany) equipped with a Sony a6000 camera (Sony, Tokyo, Japan). In addition, the natural products were stained separately to ensure that there was no birefringence.

### 2.6. Thioflavin T (ThT) Kinetic Assay

ThT fluorescence was measured at 37 °C in black 96-well plates with flat, clear bottoms, using a Tecan Spark microplate reader (Mannedorf, Switzerland). At first, Aβ_42_-peptide-containing films were dissolved in DMSO, and HEPES was added to adjust the final volume to 1000 μL. Each well contained 10 μM freshly prepared Aβ_42_ mixed with solutions of eupatorin or scutellarein at the three molar ratios (1:1, 1:5, 1:10), along with 25 μM ThT (Sigma©, St. Louis, MO, USA). The tops of the plates were sealed with microplate covers, and the fluorescence readings were performed through the bottom. A 444 nm filter was used for excitation, and a 484 nm filter was used for emission. For background measurements, ThT was diluted in HEPES. Each experiment was repeated three times, and the measurement lasted 40 h. ThT fluorescence was collected after 10 s of agitation at 270 rpm every 15 min. When using ThT concentrations higher than 5 μM, it is critical to take into consideration the background ThT fluorescence due to self-fluorescence [25]. Therefore, for the data analysis, ThT background fluorescence was subtracted from the sample readings at each time point, data were normalized on a scale of 0 to 100 arbitrary units, and the standard deviation for each point was calculated. Error bars in ThT fluorescence emission spectra represent the standard deviation of the triplicates. Data visualization was performed using the R statistical language [26] and the packages ggplot2, dplyr, ggthemes, extrafont, and ggpmisc via the integrated development environment RStudio [27].

### 2.7. In Vivo Assays in C. elegans

*C. elegans* is a small, free-living, and bacteria-eating soil nematode that typically lives for about 3 weeks at 20 °C in the laboratory. It is a nonhazardous and nonpathogenic animal that can be manipulated with standard safety rules. Several transgenic animals have been already produced and are widely used as models for AD. These animals overexpress the human Aβ peptide in their body-wall muscle cells, where the peptide gradually oligomerizes and aggregates; as a result, severe and fully penetrant age-progressive paralysis occurs. In our study, we utilized two commonly used *C. elegans* strains as models for AD, namely, CL2331 and CL4176. The former expresses the human Aβ_3-42_ peptide fused to green fluorescent protein (GFP) in its body-wall muscle cells; the animals are gradually filled with Aβ aggregates that are visible through confocal microscopy [28]. The latter strain expresses the human Aβ_42_ peptide in its body-wall muscle cells in a temperature-sensitive manner. When the temperature is upshifted, expression of the Aβ peptide is induced, resulting in paralysis of the animals within a few hours [29].

#### 2.7.1. Phenotypic Characterization

For all assays, N2 animals laid eggs for 20–30 min on nematode growth medium (NGM) plates containing either 10 μg/mL eupatorin or DMSO (control). The following phenotypic characteristics were evaluated according to standard procedures, as previously described [30,31,32]:

Pharyngeal pumping: At day 1 of adulthood, the number of pharyngeal pumps per minute was measured. Thirty-five animals per condition were scored.

Defecation assay: At day 1 of adulthood, the period in seconds from defecation to defecation (defecation cycle) was measured. Thirty-one animals per condition were scored.

Developmental timing: The duration of postembryonic development in hours from egg hatching to the L4 stage was recorded through frequent observation of the progeny. The experiment was repeated three times.

Fecundity assay: Single N2 L4 larvae were transferred onto NGM plates containing either 10 μg/mL eupatorin or DMSO. Each animal was transferred every two days to a fresh NGM plate containing fresh compound or DMSO. The progeny of each animal was scored at the L2-L3 larval stage. At least 8–10 animals per condition were scored.

Dauer formation: The progeny was maintained at 27 °C, and the number of animals at the dauer larval stage over the total number of animals was scored 72 h later. The experiment was repeated three times.

#### 2.7.2. Paralysis Assay

CL4176 animals were synchronized on NGM plates with either eupatorin (concentrations: 1, 5, 10, and 20 μg/mL) or DMSO at 16 °C for 48 h before upshifting of the temperature to 25 °C. The paralyzed animals were scored 24 h after the upshift until the paralysis of all animals. The paralysis assay was repeated three times. The criteria to score nematodes as paralyzed were (1) the presence of halos of cleared bacteria around their heads, and (2) failure to undergo half-end body wave propagation upon prodding. Animals that died during the experiment were excluded, as described in our previous work [31,33]. The log-rank (Mantel–Cox) test was used to evaluate differences between paralysis curves and to determine *p*-values for all independent data. N in the paralysis figures represents the number of paralyzed animals. Median paralysis values are expressed as the mean ± SEM.

#### 2.7.3. Confocal Analysis of Aβ Deposition

For scoring of the Aβ_3-42_ aggregates, synchronized CL2331 animals were exposed to 10 μg/mL eupatorin or DMSO and grown at 20 °C (to induce the expression of the Aβ_3-42_ peptide) until the L4 larval stage. Animals were mounted on 2% agarose pads on glass slides, anesthetized with 100 mM levamisole, and observed at room temperature using a Leica TSC SPE confocal laser scanning microscope (Leica Lasertechnik GmbH, Heidelberg, Germany). The LAS AF software (Leica Lasertechnik GmbH, Heidelberg, Germany) was used for image acquisition. Images focused in the anterior area of the nematodes were acquired with a 20x/0.70 objective. Quantification of Aβ_3-42_::GFP was conducted using ImageJ [24]. The aggregates that were quantified were the ones contained within a region of 4 square units of the surface of the animal’s body, starting from the mouth. The spots that were larger than 5 pixels were considered as aggregates. The quantification was performed automatically with the use of the function “analyze particles”. The background of each region of interest was subtracted by 5 pixels, and the threshold was set automatically using the Otsu method. Thirty animals per condition were measured.

#### 2.7.4. Statistical Analysis

The parametric two-tailed Student’s t-test was used for the comparison of the means of two groups. The log-rank (Mantel–Cox) test was used to evaluate differences between paralysis curves and to determine *p*-values for all independent data. N in the paralysis figures represents the number of paralyzed animals. The data from all assays are depicted as the average of three independent experiments (unless otherwise indicated). The median paralysis values are expressed as the mean ± SEM (shown by error bars). Asterisks denote *p*-values as follows: ** *p* < 0.01, *** *p* < 0.001, **** *p* < 0.0001, ns = not significant. Statistical analyses and graphs were produced using the GraphPad Prism 8 (GraphPad Software, Inc., San Diego, CA, USA) and Microsoft Office 365 Excel (Microsoft Corporation, Redmond, WA, USA) software packages.

### 2.8. In silico Studies of Aβ_42_ Oligomerization Inhibition

#### 2.8.1. Molecular Docking

In order to assess the binding affinity and the interaction between the oligomerized Aβ_42_ peptide and the flavones, molecular docking experiments were carried out. Particularly, protein–ligand docking was performed utilizing the publicly available software AutoDock Vina (Scripps Research, San Diego, CA, USA) [34], via the graphical user interface AutoDock Tools (ADT) [35]. Initially, the NMR-derived oligomeric structure of the Aβ_42_ peptide (including residues 17–42) was retrieved from the Protein Data Bank (PDB ID: 2BEG) [36] and imported into the molecular graphics system PyMOL [37] to segregate the first model and to remove the hydrogens of the experimentally determined structure. Afterwards, with the use of AutoDock Tools, polar hydrogens and Kollman charges were added, and the AD4 atom type was assigned to the peptide, generating the structure file in pdbqt format. The 3D structures of the ligands scutellarein and eupatorin were retrieved from PubChem [38], and their respective PubChem CIDs are 5281697 and 97214. The ligands’ structures were further processed with the use of the UCSF Chimera software [39] and the graphical interface Raccoon [40], in which Gasteiger partial charges were added and polar hydrogens were merged, resulting in the creation of the structure file for each ligand—including its active torsions—in pdbqt format. Subsequently, the proper coordinates of a rectangular parallelogram GridBox were appointed to include the whole oligomer of Aβ_42_, so that the ligands could freely bind to the most favorable region. The generated output files contained information regarding the coordinates for the first 10 conformations for each protein–ligand complex, ranked by the lowest binding affinity. Out of the 10 conformations of the complexes, the one with the lowest binding affinity was considered the most energetically favorable. Therefore, the subsequent in silico experiments were conducted on these complexes as starting points.

#### 2.8.2. Molecular Dynamics Simulations

The two docked protein–ligand complexes were subjected to molecular dynamics simulations with the use of the GROMACS software package, version 2018.1 (University of Groningen, Groningen, Netherlands) [41,42]. The topology for the Aβ_42_ peptide was generated with the all-atom additive CHARMM36 protein force field [43], which is considered to be the most suitable for protein–ligand MD simulations, whereas the ligands’ topologies were generated with the CHARMM General Force Field (CGenFF) [44]—a “drug-like” force field with the relative parameters for small compounds. The water model employed for the protein–ligand complex was TIP3P (transferable intermolecular with three points) [45]—a 3-point water model—and the systems were placed in a cubic unit cell of the same periodic distance, which was defined at 1.2 nm. In addition, the complexes were solvated with the simple point charge (SPC) [46] water model, and 5 Na+ ions were added. The solvated, neutralized systems were subjected to energy minimization runs with the steepest descent algorithm, until the maximum force reached values below 10.0 kJ/mol. Two phases of equilibration took place with the application of position restraints to the non-hydrogen atoms of the protein–ligand complex. The first phase was conducted under a constant-volume (NVT) ensemble for 100 ps, and the temperature was maintained at 310 K, with the employment of a V-rescale thermostat [47]—a modified Berendsen thermostat, suitable for protein–non-protein systems. The temperature was set at 310 K—just as in the in vitro experiments—to resemble the normal human body temperature. The second phase of equilibration was performed under a constant-pressure (NPT) ensemble for 100 ps, and the pressure was isotropically maintained at 1.013 bar (1 atm) with the use of a Berendsen barostat [48]. The equilibrated protein–ligand complexes were then set to be simulated for 500 ns at 310 K, in the absence of position restraints, while the Parrinello–Rahman barostat [49,50] was employed. Simulations were conducted with the use of the leapfrog algorithm for integration, and a 2 fs timestep was used. Long-range electrostatic interactions were modeled with the particle mesh Ewald (PME) method [51,52], while short-range non-bonded interactions were cut off at 1.2 nm. The linear constraint solver (LINCS) algorithm [53] was used for the constriction of hydrogen bonds in the entirety of the simulations, and periodic boundary conditions (PBCs) were applied in all directions.

The outputs of the simulations were analyzed with tools that are included in the GROMACS software package (University of Groningen, Groningen, Netherlands). In order to assess the potential structural changes of the oligomerized Aβ_42_ peptide, the root-mean-square deviation and the root-mean-square fluctuation were calculated by the GROMACS modules rms [54] and rmsf [55], respectively. Moreover, with the integration of the DSSP algorithm [56], graphs were generated, depicting the variation in the number of peptide residues assigned to certain secondary structural elements during the course of the simulation. Snapshots of the two protein–ligand complexes were created with the molecular visualization system UCSF Chimera, and the acquired graphs were plotted using the R statistical language [26] via RStudio (Posit, Boston, MA, USA) [27] and the packages Peptides and MDPlot.

## 3. Results

### 3.1. Computational Prediction of the Physicochemical Properties of the Natural Products

Initially, we conducted preliminary in silico research on the physicochemical properties of eupatorin and scutellarein. It is widely accepted that the early evaluation of ADMET (absorption, distribution, metabolism, excretion, and toxicity) parameters can provide useful guidelines for the preclinical stages of drug design and decrease pharmacokinetics-related clinical failures [57]. More precisely, physicochemical properties, lipophilicity, pharmacokinetics, drug-likeness, lead-likeness, and synthetic accessibility were computationally estimated using the SwissADME Web tool by the Swiss Institute of Bioinformatics (SIB) (Table S1, Supplementary File) [20]. First of all, it was predicted that eupatorin and scutellarein could be potential drug candidates, as both are characterized by drug-likeness and lead-likeness. This signifies that these plant-derived compounds have the structural and physicochemical properties of a drug and are suitable for further optimization with low synthetic difficulty. In detail, they were predicted to exhibit good aqueous solubility and high gastrointestinal absorption, which would help with oral administration. In addition, neither of the two is a substrate of P-glycoprotein—an active efflux transporter of biological membranes [58]. For these reasons, we decided to assess the inhibitory effects of these natural products on the aggregation of Aβ_42_ in vitro.

### 3.2. The Effects of the Two Natural Products on the Amyloidogenicity of Aβ_42_

**Figure 1:**
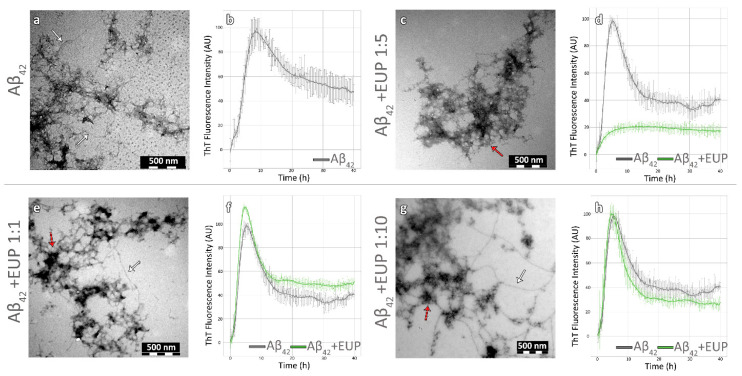
Eupatorin (EUP) delayed Aβ_42_ aggregation at a 1:5 ratio. Transmission electron micrographs of Aβ_42_ peptide alone (**a**) and co-incubated with eupatorin at ratios of 1:1 (**e**), 1:5 (**c**), and 1:10 (**g**). White arrows point to single fibrils, while red arrows point to amorphous aggregates (scale bar = 500 nm). ThT fluorescence emission spectrum of Aβ_42_ peptide as a control (**b**) and co-incubated with eupatorin for 40 h at ratios of 1:1 (**f**), 1:5 (**d**), and 1:10 (**h**). Aβ_42_ peptide and the co-incubations with eupatorin are represented by grey and green lines, respectively (Aβ_42:_ amyloid beta peptide 1-42, EUP: eupatorin).

**Figure 2:**
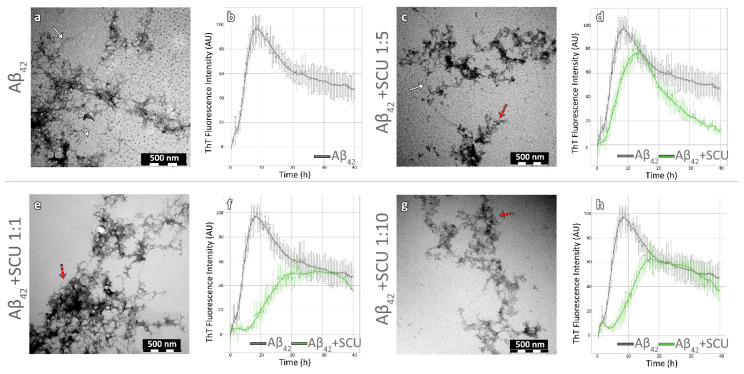
Scutellarein (SCU) was able to delay Aβ_42_ aggregation at all three concentrations. Transmission electron micrographs of Aβ_42_ peptide alone (**a**) and co-incubated with scutellarein at ratios of 1:1 (**e**), 1:5 (**c**), and 1:10 (**g**). White arrows point to single fibrils, while red arrows point to amorphous aggregates (scale bar = 500 nm). ThT fluorescence emission spectrum of Aβ_42_ peptide as a control (**b**) and co-incubated with scutellarein for 40 h at ratios of 1:1 (**f**), 1:5 (**d**), and 1:10 (**h**). Aβ_42_ peptide and the co-incubations with scutellarein are represented by grey and green lines, respectively (Aβ_42:_ amyloid beta peptide 1-42, SCU: scutellarein).

In order to examine whether eupatorin and scutellarein could inhibit or delay the amyloidogenesis of Aβ_42_, we initially incubated the peptide individually as a control. Detailed processing of electron micrographs of the control sample showed that after seven days, Aβ_42_ self-assembled into mature, unbranched fibrils of undefined length with a diameter of 8.9 nm (±1.68), as shown in Figure 1a and Figure 2a. Indeed, the ThT fluorescence measurements confirmed that amyloid-like fibrils had formed at approximately eight hours (Figure 1b and Figure 2b). It is worth noting that the lag phase was approximately an hour and a half, but we have also observed this before in one of our previous works [59]. Moreover, gel containing amyloid-like fibrils was stained with Congo red dye and observed under crossed polars of a polarized microscope. Congo red staining and polarized microscopy are among the first criteria to determine the amyloid properties of a protein in vitro or in histochemical studies, along with TEM. In the bright field, we observed that the sample was stained red, indicating that Congo red specifically binds to the sample, while under polarized light we observed the characteristic apple-green birefringence that amyloids typically exhibit, confirming the amyloid nature of the sample, as shown in Figure S2 in the Supplementary File. Of course, the Congo red staining is not as indicative as TEM for the identification of amyloid-like fibrils, but it is an amyloid-specific dye like thioflavin T, owing to its orientation between the beta-strands of amyloid fibers [60].

In the presence of eupatorin, Aβ_42_ aggregation was significantly reduced according to the TEM observations. To be more specific, the best results were recorded at the ratios of 1:5 and 1:10. The grids were barely empty, hardly any fibrils were observed, and the dominant morphology was amorphous and prefibrillar aggregates (Figure 1c,g). These results are consistent with the absence of apple-green birefringence after Congo red staining (Figure S3, Supplementary File). Furthermore, the effect of eupatorin was evident in the ThT kinetic assays (Figure 1d,f,h). At the 1:5 ratio, a sharp reduction in ThT fluorescence was already recorded in the first five hours (Figure 1d). Concerning the ratio of 1:1, the fibrils’ morphology appeared no different than that of Aβ_42_ alone. In Figure 1e, it is apparent that the peptide aggregated into amyloid-like fibrils and amorphous aggregates (Figure 1e, white and red arrows, respectively). More specifically, we observed elongated and unbranched fibrils of indefinite length that formed dense networks, and their diameter ranged from 8 to 9 nm (Figure 1e, white arrow). Lastly, only in this concentration did we record a light yellow–green birefringence, as expected based on the number of amyloid-like fibrils formed (Figure S3, Supplementary File). Given these results, we concluded that eupatorin caused a dose-dependent reduction in Aβ_42_ aggregation.

When incubated with scutellarein, the number of fibrils decreased as the concentration of the compound increased. It is worth mentioning that the dominant morphology was not a network of amyloid-like fibrils, but amorphous aggregates at all three concentrations (red arrows in Figure 2c,e,g). In addition, the diameter of the fibrils was found to be 8.2 nm (±0.8) at the 1:1 ratio, 7.1 nm (±0.2) at the 1:5 ratio, and 7.8 nm (±1.1) at the 1:10 ratio (Figure 2c,d,g, white arrows). The TEM results were consistent with the ThT kinetic assays shown in Figure 2d,f,h, where a decrease in the maximal fluorescence signal by 34.2%, 12.09%, and 43.2% was measured at ratios of 1:1, 1:5, and 1:10, respectively. The maximum signal was measured after 16 h—twice as long compared to Aβ_42_. Furthermore, we observed a longer lag phase and a slower exponential phase in contrast to the control. Finally, the amorphous aggregates and the reduced number of amyloid-like fibrils could explain the absence of apple-green birefringence when droplets from the co-incubation samples were stained with Congo red and observed under crossed poles of the polarizing microscope (Figure S3, Supplementary File).

### 3.3. The Impact of Each Natural Compound on the Tertiary Structural Stability of the Aβ_42_ Oligomer

The results of molecular docking suggested that both compounds show good binding affinity, forming thermodynamically favorable complexes with the oligomer of Aβ_42_ peptide. The lowest binding affinities for the peptide in complex with scutellarein and eupatorin exhibited values of −6.8 and −6.2 kcal/mol, respectively. These complexes were subsequently used for the conduction of molecular dynamics simulations. Among the simulated data analyzed, those considered to be of utmost importance were the number of residues participating in certain secondary structural elements of the Aβ_42_ peptide—primarily beta-sheets and helices—and the possible change of the Aβ_42_ peptide conformation by assessing the root-mean-square deviation (RMSD) and the root-mean-square fluctuation (RMSF) of the Aβ_42_ peptide chains that form its oligomeric state.

**Figure 3:**
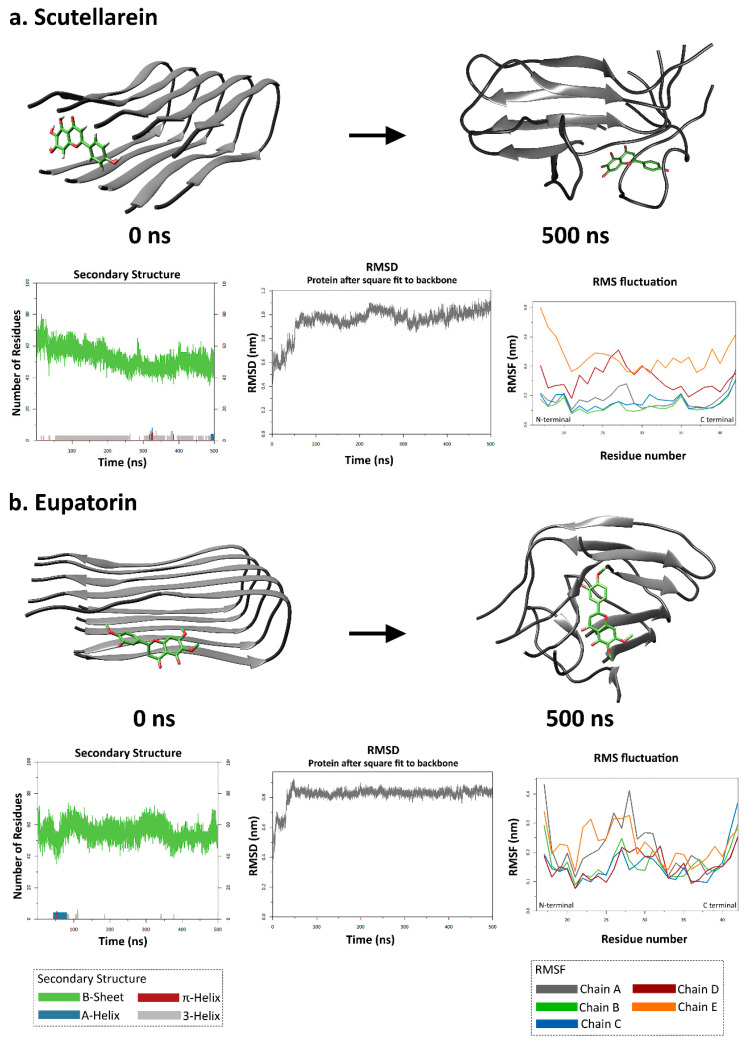
Results of molecular dynamic simulations of Aβ_42_ oligomer with scutellarein (**a**) and eupatorin (**b**). Both compounds caused a decrease in the number of amino acids involved in beta-strands. Simulation frames of the Aβ_42_ oligomer at 0 ns and 500 ns. The Aβ_42_ peptide is colored grey, and the inhibitors are light green. The first plot for each natural compound corresponds to the number of residues assigned to the B-sheet (green), A-helix (blue), π-helix (red), and 3-helix (grey) during the simulation, according to the DSSP calculations. The second plot depicts the root-mean-square deviation (RMSD) of the Aβ_42_ oligomer during the course of the simulation. The root-mean-square fluctuation (RMSF) per residue for the five chains of the Aβ_42_ oligomer is depicted in the third plot (RMSF: root-mean-square fluctuation, RMSD: root-mean-square deviation).

As far as the secondary structure is concerned, all compounds caused a decrease to some extent in the number of amino acid residues of the Aβ_42_ peptide involved in beta-strands—which is inextricably linked to its pathogenic character—and, albeit temporarily, the formation of helices. Scutellarein triggered a steep decrease in the number of beta-strands, with the number of residues in strands reduced by 23% when comparing the average numbers between the first and the last 5 ns of the simulation. Eupatorin followed in efficacy, with a stable decreasing tendency (12% reduction in the number of residues in beta-strands). Subsequently, the oligomer’s conformational changes after ligand binding and the course of the simulation were evaluated by calculating the RMSD. Higher RMSD values indicate that the interaction between the compounds and the Aβ_42_ oligomer leads to a less stable structure, proving their potency. As shown in Figure 3, scutellarein and eupatorin caused mild conformational changes, with maximum values around 1 nm.

RMSF analysis was employed to assess the flexibility of the peptide structure per amino acid residue and showed that ligand binding induces conformational changes to a greater extent in the C-termini of the peptide chains that form the oligomer. The complex with eupatorin exhibited less fluctuation along the entire length of all chains as compared to the other ligand, without exceeding 0.4 nm (Figure 3b). As far as the Aβ_42_ peptide–scutellarein complex is concerned, the chains that underwent substantial changes in their structural integrity—exhibiting higher mobility and being more flexible at the end of the molecular dynamic simulation—were D and E. The N-termini of these two chains showed greater fluctuation compared to the C-termini, and the effect of scutellarein was evident for all of the residues of chains D and E. However, chains A, B, and C were characterized by lower RMSF values, meaning that they were less flexible and were not extremely affected by their interaction with scutellarein (Figure 3a).

### 3.4. The Effect of Eupatorin on C. elegans

Since our in vitro and in silico results confirmed that both flavones are able to prevent the fibrillogenesis of Aβ_42_, we proceeded with in vivo experiments on specific strains of *C. elegans* that are commonly used as animal models for Aβ accumulation and AD [28]. Since the anti-amyloid properties of scutellarein have already been investigated in vivo by Gea-Gonzales et al. [61], we conducted in vivo assays with *C. elegans* only for eupatorin, which had never been tested before. 

**Figure 4:**
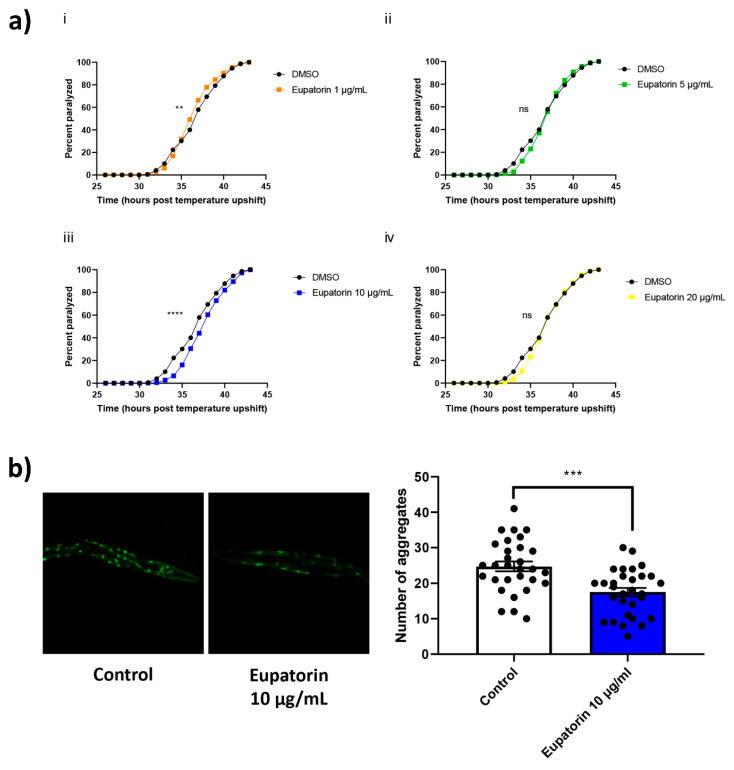
Treatment with eupatorin confers protection against Aβ toxicity. (**a**) Paralysis curves of CL4176 animals treated with various concentrations of eupatorin or DMSO (control). Control: mean = 36.67 ± 1.33, n = 947; (**i**) 1 μg/mL eupatorin: mean = 36.67 ± 0.88, n = 890, ** *p* < 0.01; (**ii**) 5 μg/mL eupatorin: mean = 37.33 ± 0.67, n = 959, ns; (**iii**) 10 μg/mL eupatorin: mean = 37.67 ± 0.88, n = 901, **** *p* < 0.001; and (**iv**) 20 μg/mL eupatorin: mean = 37.33 ± 0.67, n = 870, ns, 3 independent experiments; ns: not significant. Median paralysis values are expressed as the mean ± SEM. N denotes the number of animals that were paralyzed. Curves are the pooled result of the indicated independent experiments. For paralysis experiments, the log-rank Mantel–Cox test was used. (**b**) Representative fluorescence micrographs of CL2331 animals expressing the human Aβ_3-42_ peptide conjugated to GFP treated with 10 μg/mL eupatorin (n = 30) or DMSO (control; n = 30) from egg hatching throughout the experiment, and the relative quantification of amyloid deposits in the anterior area of the animals. Images were acquired with a 20×/0.70 objective, three independent experiments. N represents the number of animals quantified. Each dot on the figure represents a quantified animal. The values are reported as the mean ± SEM; *** *p* < 0.001 (two-tailed Student’s *t*-test).

To evaluate the potential of eupatorin to confer protection against Aβ toxicity, we took advantage of a transgenic *C. elegans* strain (CL4176) that expresses the human Aβ_42_ peptide in its body-wall muscle cells in a temperature-sensitive manner. When the temperature is upshifted, expression of Aβ peptide is induced, resulting in paralysis of the animals within a few hours [29]. We tested various concentrations of eupatorin ranging from 1 to 20 μg/mL; 10 μg/mL was the concentration that produced positive results (Figure 4a). Animals treated with eupatorin were paralyzed significantly later as compared to the control animals.

We sought to investigate whether this decelerated paralysis was due to lower levels of Aβ aggregates. We therefore took advantage of the CL2331 strain. Treatment with eupatorin resulted in a reduced number of Aβ aggregates (Figure 4b). In total, our results revealed a protective effect of eupatorin against Aβ toxicity.

*C. elegans* is an increasingly used model for toxicity testing, as it has various phenotypic characteristics that change upon toxic exposure [62]. We therefore evaluated the following phenotypic characteristics in wild-type nematodes in the presence of 10 μg/mL eupatorin and in control animals (treated with DMSO): developmental time, fertility, egg lethality, pharyngeal pumping, defecation rate, and dauer formation (Table 1). Phenotypic characteristics were found to be unaltered, with the exception of pharyngeal pumping, which was found to be significantly increased upon treatment with eupatorin. This increase was considered to be a positive outcome, since it has been associated with enhanced organismal healthspan [30,63,64]. In total, our results advocate for low eupatorin-dependent toxicity.

## 4. Discussion

In this study, we attempted for the first time to elucidate the mechanism of action of two rare flavones—namely, eupatorin and scutellarein—on the aggregation of Aβ_42_. Since many natural products today are tested as drugs, we posed a question about the drug-likeness of our compounds. Having established that, we conducted dose-dependent experiments to investigate whether these compounds could inhibit or at least delay the aggregation of the Aβ_42_ peptide in vitro.

To begin with, the in silico evaluation of the structural and pharmacokinetic properties of the compounds led to the conclusion that they satisfy most of the criteria of drug candidates [65]. Regarding the in vitro results, both compounds were effective, since they prevented Aβ_42_ from self-assembling into amyloid-like fibrils. This observation was verified by a decrease in the ThT fluorescence intensity at the 1:5 ratio in the presence of eupatorin, and at all ratios in the presence of scutellarein. Moreover, these results were confirmed by the absence of apple-green birefringence after Congo red staining. Consequently, we suggest that eupatorin and scutellarein are able to disrupt the aggregation of Aβ_42_. 

Previous studies have demonstrated the anti-inflammatory, antioxidant, neuroprotective, and metal-chelating properties of scutellarein, which has been used as a scaffold for the development of multifunctional ligands for the treatment of Alzheimer’s disease [66,67,68]. Recent studies confirm our in vitro results and show that scutellarein is able to extend the lifespan of the CL2355 strain (a strain with pan-neuronal expression of the human Aβ peptide [69]), while the number of Aβ_42_ aggregates was found to be significantly reduced in the CL2331 strain [61]. For this reason, we focused our in vivo experiments on eupatorin, which had never been evaluated before. Concerning the effect of this compound as anti-amyloid agent in the two AD nematode models, our data indicate that this flavone can significantly reduce the number of Aβ aggregates and decelerate the paralysis that occurs upon accumulation of human Aβ peptide.

Our experimental results seemed to be consistent with those of molecular dynamic simulations, as both compounds caused noticeable alterations to the conformation of the Aβ_42_ oligomer when comparing the 0 ns frames to the 500 ns frames. In particular, eupatorin contributed to the decrease in the number of residues in beta-strands, while a significant increase in the number of residues in helices was observed. Additionally, what stood out in the RMSF analysis was the flexibility of the hydrophilic N-terminus and the hydrophobic C-terminus at the end of the simulation. Prior studies have already noted the importance of the C-terminus in Aβ stability, as it is said to initiate the conformational change from α-helix to β-sheet and promote nucleation [70,71].

Our results, in addition to those of previous studies on other complementary biological properties of scutellarein and eupatorin, show that both are good candidates for the development of multifunctional Alzheimer’s-disease-modifying agents. These are flavone aglycones and are seldom found in that form in natural sources; they are part of glycosides in plants, e.g., scutellarin. When scutellarin is ingested by humans, it is hydrolyzed into aglycones in the colon and is then absorbed as scutellarein, so scutellarein might be the real bioactive component in the body. Oral administration of scutellarein to rats showed that scutellarein had better neuroprotective effects than scutellarin, and it could attenuate neuronal injury by ischemia/reperfusion [72] and focal cerebral occlusion/reperfusion [73].

Concerning the blood–brain barrier distribution, several flavones have been found to be able to pass through it, such as genistein, isoliquiritigenin, and kaempherol [74,75]. In the future, we will aim to assess the BBB permeability of scutellarein and eupatorin by utilizing CaCo-2 and BBB cell models that have already been used by other groups to explore flavones’ properties. In addition, further optimization could possibly enhance the pharmacokinetic properties of the compounds.

## 5. Conclusions

In this integrated study, we assessed the inhibitory effects of scutellarein and eupatorin on the aggregation of the Aβ_42_ peptide in vitro, in silico, and in vivo. According to the experimental results and the molecular dynamics simulations, eupatorin exhibited an encouraging inhibitory effect on Aβ_42_ amyloidogenesis. In the future, we suggest that this 5,3′-dihydroxy-6,7,4′-trimethoxyflavone or other optimized compounds with similar structures should be further tested and optimized as potential drug candidates against AD.

## Figures and Tables

**Table 1:** Phenotypic analysis of wild-type animals treated with 10 μg/mL eupatorin or the diluent (DMSO; control).

	Control	10 μg/mL Eupatorin
Developmental time ^1^	51.27 ± 0.12	51.15 ± 0.18
Fertility ^2^	269.8 ± 11.81	243.12 ± 7.72
Egg lethality ^3^	0	0
Pharyngeal pumping ^4^	279.4 ± 3.06	299.3 ± 2.17 ****
Defecation ^5^	47.45 ± 0.71	48.26 ± 1.33
Dauer formation ^6^	0.51± 0.07	0.52 ± 0.08

All assays were performed at 20 °C unless noted otherwise. ^1^ Duration of postembryonic development (hours from egg hatching to L4 stage). ^2^ Number of offspring per worm. ^3^ Number of eggs that did not hatch. ^4^ Pumps in 1 min at day 1 of adulthood. ^5^ Duration of defecation cycle in seconds at day 1 of adulthood. ^6^ Percentage of animals becoming dauer larvae at 27 °C. Error bars denote the mean ± SEM; p denotes the *p*-value of Student’s *t*-test, **** *p* < 0.0001.

## Data Availability

Data available within the article or Supplementary Materials.

## Supplementary Materials

The following supporting information can be downloaded at: https://www.mdpi.com/article/10.3390/biomedicines11051357/s1, Figure S1: Flavone numbering. Figure S2: Photomicrographs of Congo-red-stained gel derived from the incubation of Aβ_42_ peptide. The Congo red dye was bound, as seen under bright-field illumination (up). Apple-green birefringence was observed under crossed poles (down). Figure S3: Photomicrographs of Congo-red-stained gels derived from the co-incubation of Aβ_42_ peptide at three ratios with the natural products eupatorin (1:1(1b), 1:5(1c), 1:10(1d)) and scutellarein (1:1(2b), 1:5(2c), 1:10(2d)). The natural products were stained separately to ensure that there was no birefringence (1a, 2a). The Congo red dye was bound, as seen under bright-field illumination (upper). No apple-green birefringence was observed under crossed poles at any concentration. Table S1: Prediction of physicochemical properties, pharmacokinetics, and drug-likeness of eupatorin and scutellarein using SwissADME.

## Abbreviations

AD	Alzheimer’s disease
Aβ	Amyloid beta
APP	Amyloid precursor protein
ADT	AutoDock Tools
CGenff	CHARMM General Force Field
DMSO	Dimethyl sulfoxide
GFP	Green fluorescent protein
LINCS	Linear constraint solver
MD	Molecular dynamics
NGM	Nematode growth medium
PME	Particle mesh Ewald
PBC	Periodic boundary condition
RMSD	Root-mean-square deviation
RMSF	Root-mean-square fluctuation
SPC	Simple point charge
SIB	Swiss Institute of Bioinformatics
ThT	Thioflavin T
TEM	Transmission electron microscopy
TIP3P	Transferable intermolecular with three points
HFIP	1,1,1,3,3,3-Hexafluoro-2-propanol
HEPES	4-(2-Hydroxyethyl)-1-piperazineethanesulfonic acid
