# Supplementary material for: A Multilevel Study of Eupatorin and Scutellarein as Anti-Amyloid Agents in Alzheimer’s Disease
Source: Biomedicines. 2023 May 4;11(5):1357. doi: 10.3390/biomedicines11051357 (PMC10216506; doi:10.3390/biomedicines11051357)
Supplement: Supplementary file 1 [file biomedicines-11-01357-s001.zip › biomedicines-2349601-supplementary.pdf]

Supplementary File

A Multilevel Study of Eupatorin and Scutellarein as Anti-Amyloid Agents in Alzheimer's Disease

Aikaterini E. I. Rizou, Georgia I. Nasi, Yiorgos Paikopoulos, Dimitra S. Bezantakou, Konstantina D. Vraila, Panagiotis M. Spatharas, Virginia Dimaki, Nikos C. Papandreou, Fotini N. Lamari, Niki Chondrogianni and Vassiliki A. Iconomidou

Table S1. Prediction of physicochemical properties, pharmacokinetics and druglikeness of eupatorin and scutellarein using SwissADME.

Properties	Eupatorin	Scutellarein
Structure	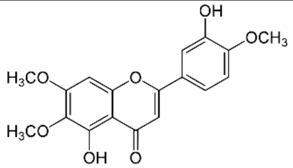	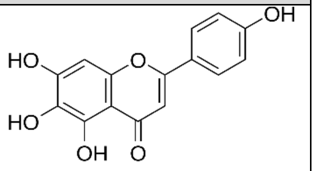
**Physicochemical Properties**		
Molecular Formula	$C_{18}H_{16}O_7$	$C_{15}H_{10}O_6$
Molecular Weight (g/mol)	344.32	286.24
Number of rotatable bonds	4	1
Number of heavy atoms	25	21
Number of aromatic heavy atoms	16	16
Fraction Csp3	0.17	0
Number of H-bond acceptors	7	6
Number of H-bond donors	2	4
Topological Polar Surface Area (A^2)	98.36	111.13
**Lipophilicity**		
Log $P_{o/w}$	2.53	1.81
Water solubility	Moderately soluble	Soluble
**Pharmacokinetics**		
Gastrointestinal absorption	High	High
Blood-Brain barrier	No	No
P-glycoprotein substrate	No	No
CYP1A2 inhibitor	Yes	Yes
CYP2C19 inhibitor	No	No
CYP2C9 inhibitor	Yes	No
CYP2D6 inhibitor	Yes	Yes
CYP3A4 inhibitor	Yes	Yes
Log Kp (skin permeation) (cm/s)	-5.99	-6.16
**Druglikeness**		
Lipinski	Yes; 0 violation	Yes; 0 violation
Ghose	Yes	Yes
Veber	Yes	Yes
Egan	Yes	Yes

Muegge	Yes	Yes
Bioavailability Score	0.55	0.55
**Medicinal Chemistry**		
Leadlikeness	Yes	Yes
Synthetic accessibility	3.43	3.04

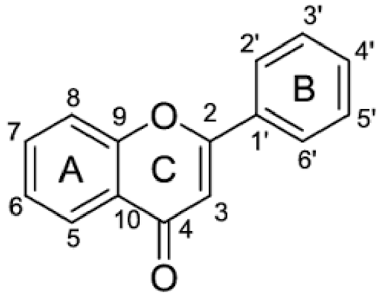

Figure S1. The flavone numbering.

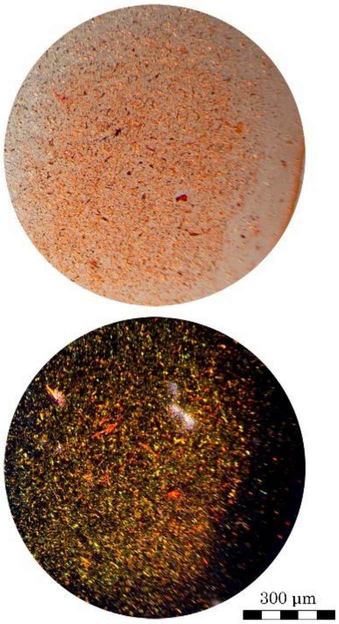

Figure S2. Photomicrographs of Congo Red-stained gels derived from the incubation of Aβ_{42} peptide as control. The Congo Red dye is bound, as seen under bright field illumination (up). Apple-green birefringence was observed under crossed polars (down).

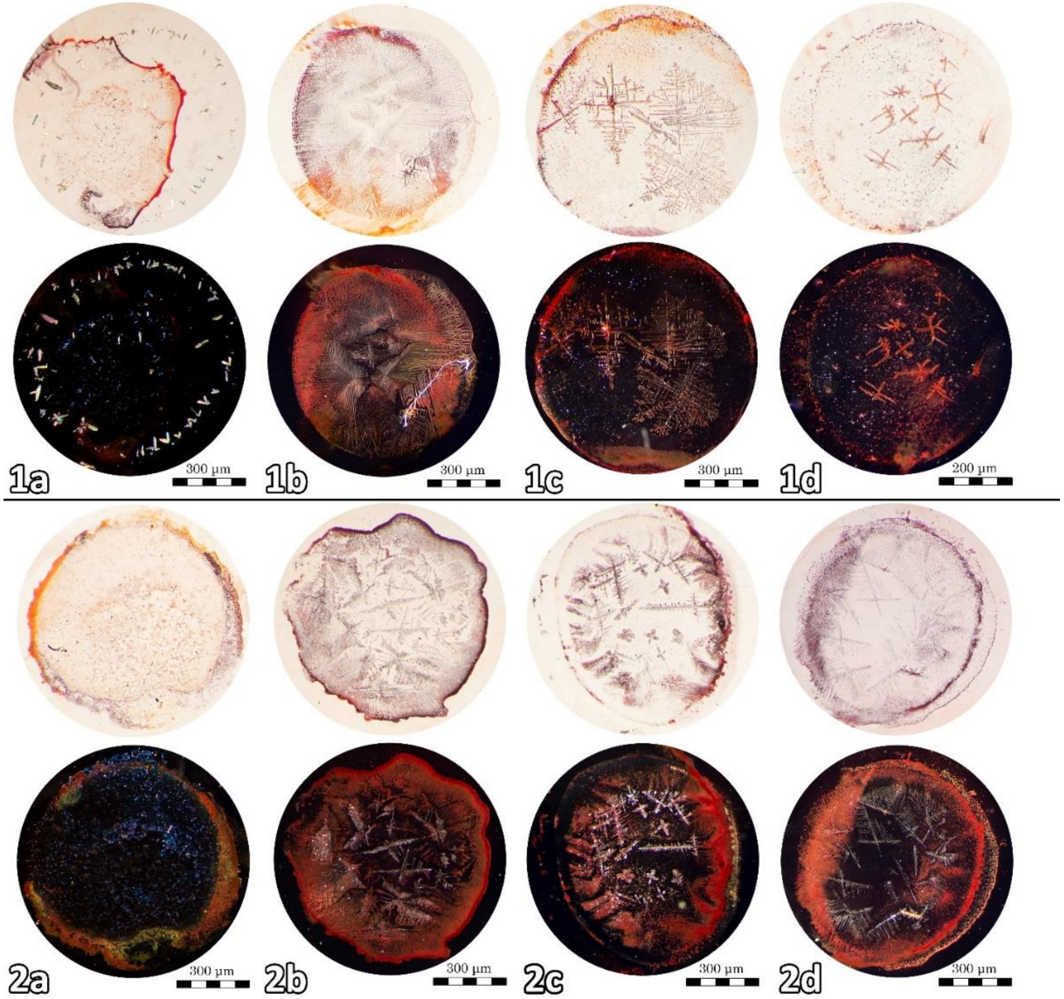

Figure S3. Photomicrographs of Congo Red-stained gels derived from the co-incubation of Aβ_{42} peptide in three ratios with the natural products, eupatorin (1:1(1b), 1:5(1c), 1:10(1d)), scutellarein (1:1(2b), 1:5(2c), 1:10(2d)). The natural products were stained separately to control that there was no birefringence (1a, 2a). The Congo Red dye is bound, as seen under bright field illumination (upper). No apple-green birefringence was observed under crossed polars in any concentration.
